# Osteoimmunology-Driven Design of Dental Implant Materials: From Immune Response to Osseointegration

**DOI:** 10.3390/ma19122627

**Published:** 2026-06-18

**Authors:** Julia Kloc, Kinga Janusiewicz, Karolina Jędrzejczyk, Agnieszka Kijora, Aleksandra Jankowska, Marcelina Księżopolska-Markiewicz, Weronika Pająk, Jakub Kleinrok, Jacek Baj

**Affiliations:** 1 Department of Forensic Medicine, Medical University of Lublin, Jaczewskiego 8b, 20-090 Lublin, Poland; klocjulia@gmail.com (J.K.); janusiewiczkinga@gmail.com (K.J.); kjedrzejczyk445@gmail.com (K.J.); kijora.agnieszka@gmail.com (A.K.); jankowskaa2002@gmail.com (A.J.); 2 Department of Correct, Clinical and Imaging Anatomy, Medical University of Lublin, Jaczewskiego 4, 20-090 Lublin, Poland; mpksiezopolska@gmail.com (M.K.-M.); jacek.baj@umlub.edu.pl (J.B.); 3 Department of Clinical Pathomorphology, Medical University of Lublin, Jaczewskiego 8b, 20-090 Lublin, Poland

**Keywords:** osteoimmunology, dental implants, biomaterials, immune response, immunomodulatory materials, additive manufacturing

## Abstract

The success of dental implantation depends on both mechanical stability and the host’s immune response to the implanted biomaterials. Osteoimmunology emphasizes that early immune responses at the implant-tissue interface are critical for bone healing and long-term osseointegration. The immune response primarily consists of immune cells, particularly macrophages, neutrophils, and lymphocytes, which interact with osteogenic cells through cytokine networks and signalling pathways, such as RANK/RANKL/OPG. Additionally, it modulates both bone formation and resorption. This review focuses on summarizing the mechanisms that shape the immune response around implants by dental implant materials. It describes mechanisms related to bulk composition, surface topography, and mechanical properties, and highlights macrophage polarization and the transition from inflammation to regeneration. The review discusses current immunomodulatory strategies, including bioactive surfaces, ion doping, nanopatterning, drug-releasing surfaces, and responsive materials, as well as advances enabled by additive manufacturing. The review also discusses experimental models used to study osteoimmunological interactions and the clinical significance of immune dysregulation in peri-implant diseases. The design of biomaterials based on osteoimmunology represents a shift toward immune-compatible implants that aim to improve regenerative outcomes and long-term implant success.

## 1. Introduction

Dental implants are now widely recognized as the gold standard in the restoration of missing teeth, as they provide patients with highly predictable functional and aesthetic outcomes, providing patients with highly predictable functional and aesthetic outcomes. Long-term studies estimate the 10-year survival rate at approximately 96.4% [1,2,3,4]. More recently, extensive clinical meta-analyses have demonstrated that clinical success varies among materials; while commercially pure titanium [Ti] exhibits a survival rate of 97.7%, titanium–zirconium [Ti-Zr] alloys achieve the highest clinical success at 98.6%, significantly outperforming zirconia [Zr] implants (93.8%) [3,5,6]. Traditionally, the clinical success of implant treatment has been attributed to the process of osseointegration. It was, which was originally defined as the formation of a direct, structural, and functional connection between living bone and the loaded surface of an artificial implant [7,8,9,10,11,12]. For decades, the classical paradigm of biomaterial design focused almost exclusively on stimulating bone-forming cells, osteoblasts (OBs), to achieve mechanical stability, based on the assumption that the material should be biologically inert [5,7,13,14,15].

However, it should be noted that the implant placement procedure itself is inherently associated with tissue trauma, which immediately triggers an immune response to the foreign body [7,9,14]. It is now believed that successful osseointegration depends equally on the active management of this early inflammatory response, shifting the understanding of osseointegration toward a state of foreign body equilibrium (FBE) [7,8,9,10,13,16,17]. This has led to the development of “osteoimmunology,” an interdisciplinary field of medicine that studies the highly complex and dynamic communication between the skeletal and immune systems [7,8,14,18,19,20]. It is widely accepted that the early response of immune cells at the implant-tissue interface directly determines subsequent processes of osteogenesis and angiogenesis and long-term implant survival [2,7,13,14,15]. Consequently, the classical understanding of osseointegration is increasingly evolving toward the broader concept of “osteoimmunological integration” (OII), denoting a target state of equilibrium between the foreign biomaterial and the host’s immune system [8,16].

The local immune microenvironment around the implant is shaped by various subpopulations of immune cells, among which neutrophils, macrophages, and lymphocytes play a key role [7,8,9]. Neutrophils are the first cells to migrate to the site of tissue damage. They are responsible for wound clearance and secrete inflammatory mediators, which subsequently recruit other immune cells. Moreover, they can also release neutrophil extracellular traps (NETs), which, if unregulated, may impair the healing process [7,9,18,21]. However, the main drivers of the regeneration process are macrophages. They are characterized by immense plasticity and the ability to polarize into two opposing phenotypes: the classically activated pro-inflammatory M1 phenotype and the alternatively activated pro-regenerative M2 phenotype [7,8,13,14]. The early phase of the response to the biomaterial is dominated by M1 macrophages, which secrete cytokines such as TNF-α, IL-1β, and IL-6 to eliminate contaminants and stimulate the early stages of healing [7,8,9,13,14,15]. To prevent chronic inflammation, which could lead to fibrous encapsulation of the implant and bone destruction, a timely switch in polarization from the M1 to the M2 phenotype is essential [7,8,14]. M2 macrophages suppress inflammation and secrete anti-inflammatory cytokines (e.g., IL-10) and potent growth factors (including TGF-β, bone morphogenetic protein-2 (BMP-2), and vascular endothelial growth factor), which directly promote angiogenesis and the differentiation of mesenchymal stem cells (MSCs) into OBs [2,7,13,14,22]. T lymphocytes, including regulatory T cells (Tregs), also play a significant role in controlling this homeostasis. They suppress excessive inflammatory responses mediated by Th17 cells and help to minimize bone resorption in pathological conditions such as peri-implantitis [7,8,14,18,22,23,24].

Immune system cells communicate with osteogenic cells and osteoclasts (OCs) through a network of cytokines and intracellular signaling pathways, among which the Receptor Activator of Nuclear Factor κB (RANK)/receptor activator of nuclear factor κ-B ligand (RANKL)/Osteoprotegerin (OPG) axis plays a fundamental role [7,8,13,14,19,21,23,25,26,27]. The RANKL secreted by OB and Th17 lymphocytes binds to the RANK on the surface of OC precursors, directly stimulating their maturation and leading to bone resorption [7,8,13,18,25,26]. A natural inhibitor of this process is OPG, a soluble trap receptor that competitively binds RANKL, thereby preventing OC activation [7,8,13,15,18,25]. Low RANKL/OPG ratio in the peri-implant microenvironment is crucial for inhibiting excessive osteoclastogenesis and ensuring that bone-forming processes prevail [7,8,13,14,21,23,26].

A deep understanding of these biological interactions has led to the design of innovative dental biomaterials into the phase of “osteoimmunomodulation” (OIM)—the deliberate modification of surfaces to program the immune system toward a reparative response [2,13,15,25,27]. Modifying the physicochemical properties of titanium surfaces, including their micro- and nanotopography and wettability, can directly modulate macrophage behavior, accelerating their transition to the M2 phenotype [9,17,25,28,29,30]. Contemporary strategies also utilize the doping of materials with active ions; for example, the presence of strontium (Sr), magnesium (Mg), or copper (Cu) ions exhibits a dual therapeutic effect, stimulating OBs and the M2 macrophage phenotype while simultaneously inhibiting OCs and exhibiting long-term antibacterial properties [1,13,14,27,31,32,33,34]. An extremely promising direction involves nanostructures (e.g., titanium dioxide nanotubes or quaternized silicon carbon nitride (QSiCN) coatings) serving as platforms for the localized, controlled release of drugs or antimicrobial substances, which protects tissues from microbial invasion and minimizes inflammation [1,2,14,34,35]. This “fit-and-forget” approach protects tissues from microbiological invasion, manages biofilm formation and minimizes inflammation without compromising tissue integration [1,10,33,34,36,37].

A vital research on OII was the incorporation of additive manufacturing (AM) methods (3D printing), such as selective laser melting (SLM), which allows for the creation of porous implants with precise geometries [1,10,14,38,39,40]. Such three-dimensional structures exhibit excellent interaction with various cell types within the oral cavity, stimulating, among other things, increased secretion of collagen and angiogenic markers, and promoting the expression of M2 cytokines in monocytes, which makes them highly biocompatible [10,14,25,38,39].

This review article focuses on summarizing the mechanisms through which advanced implant materials in dentistry shape the local immune response. In particular, the study describes the influence of overall composition, topography, and mechanical properties on macrophage polarization and the transition from the inflammatory to the regenerative phase.

Furthermore, it discusses in detail current OIM (osteoimmunomodulatory) strategies, including surface modifications, ionic doping, nanoporous engineering, drug-releasing surfaces, and the latest possibilities offered by 3D printing. The review also covers various experimental models (in vitro and in vivo) used to evaluate biomaterials in the context of osteoimmunology and discusses the clinical significance of immune disorders in patients predisposed to peri-implant diseases. While several recent reviews have discussed surface modifications in implant dentistry, they predominantly focus on traditional osseointegration parameters, broad antimicrobial properties, or general clinical survival rates [3,4,6,10,12,29]. Concurrently, emerging literature has begun to explore the role of the host immune system, examining topography-mediated immunomodulation or the specific regulation of innate immunity by implant surface micro-roughness and hydrophilicity [3,5,29].

However, the present review provides a distinct perspective by bridging the gap between fundamental molecular osteoimmunology and cutting-edge clinical translatability. Unlike previously published works, this manuscript critically evaluates the synergistic potential of AM (3D printing) and smart, ion-doped nanocoatings in actively programming macrophage M1/M2 polarization [34,38]. Furthermore, it uniquely addresses critical but often overlooked transitional barriers, such as the discrepancy between simplified 2D in vitro models and complex 3D clinical implant geometries, as well as the detrimental effects of routine clinical sterilization and storage on sensitive nanocoating [1,38]. By synthesizing these cellular, engineering and clinical dimensions, this review establishes a comprehensive framework for OII and outlines specific, testable future directions, clearly setting it apart from existing literature in the field.

## 2. Search Strategy

A structured literature search was conducted to identify studies evaluating the immune response and osseointegration of dental implants. The search was performed in the PubMed/MEDLINE, ResearchGate, and Google Scholar databases. The following keywords and their combinations were applied: “osteoimmunology”, “immune modulation”, “dental implants”, “biomaterials”, “additive manufacturing”.

To ensure the relevance and recency of our findings, we restricted the reviews to English-language papers published between 2016 and 2026. We searched for studies that specifically examined the impact of dental implant materials, surface modifications such as coatings, ion doping, and topography changes, on local immune reactions and osseointegration, or AM techniques on the local immune response and subsequent osseointegration. We focused on studies tracking key immunological markers, including macrophage polarization (M1/M2 phenotype transition), cytokine profiling (e.g., TNF-α, IL-10), or the RANK/ RANKL/ OPG signaling pathway. We qualified original in vitro studies, in vivo animal models, human clinical trials, and comprehensive review articles.

On the other hand, we excluded older publications, non-English texts, and non-peer-reviewed materials like preprints, editorials, and letters to the editor. We also passed over case reports, news articles, marketing content, manufacturer materials, and studies strictly dedicated to orthopedic implants with no connection with dental or maxillofacial applications. Undoubtedly, papers evaluating only the mechanical, structural, or physical features of biomaterials—without any biological or immunological evaluation—were counted out.

The searching and evaluating process was iterative. After a preliminary review of the titles and abstracts, we analyzed the full texts to confirm their eligibility according to the previously mentioned criteria. Additional relevant publications were identified by combing through the reference lists of the selected articles. Moreover, papers reporting on macroscopic and clinical inflammatory indications (such as bleeding on probing or fibrous tissue encapsulation) as well as clinical survival rates of modified biomaterials were included to associate cellular osteoimmunological mechanisms with clinical outcomes.

### Quality Assessment and Risk of Bias

As this manuscript is structured as a comprehensive narrative review, a formal quantitative risk of bias assessment (e.g., using standardized Cochrane or SYRCLE tools) was not uniformly applied to individual studies. However, a critical qualitative appraisal of the included literature reveals several consistent methodological trends. Regarding sample sizes and study design, while the cited clinical meta-analyses evaluate robust patient cohorts to confirm high long-term implant survival rates (e.g., 96.4% over 10 years), the primary in vitro and in vivo investigations of novel immunomodulatory nanocoatings frequently rely on limited sample sizes and small animal models. This restricts the statistical power and direct clinical extrapolation of their findings. Furthermore, the reproducibility of the reported osteoimmunological outcomes faces significant translational barriers. Many included laboratory studies utilize simplified, 2D flat Ti discs that fail to accurately reflect the complex, 3D threaded geometries and dynamic mechanical environments of clinical implants.

## 3. Osteoimmunology at the Implant Interface

Recent advances in bone immunology research have transformed the understanding of osseointegration, redefining it as a process dependent on the immune system, which regulates bone regeneration at the implant interface. Historically considered a passive bone response, it is now understood as the result of dynamic interactions between biomaterials and host immune cells [16]. In this sense, implant placement is intended to induce a controlled immune response that establishes an FBE, where pro-inflammatory and anti-inflammatory signals are balanced to promote tissue healing [16,41]. A recent study using quantitative polymerase chain reaction (qPCR) and histological analysis found that numerous RNA markers associated with the immune system and inflammation in tissues adjacent to titanium implants were selectively upregulated during the 1–4-week observation period. This finding further supports the relationship between immune responses and osseointegration [41].

### 3.1. Key Immune and Bone Cells

Bone tissue is primarily composed of OB, OCs, and osteocytes, whose coordinated activity maintains skeletal homeostasis [13]. OBs originate from skeletal stem cells (SSCs), a subset of the MSC lineage that resides in the bone marrow. SSCs can then differentiate into other skeletal cell types, namely chondrocytes, adipocytes, and OBs [19]. The latter are responsible for bone formation by deposition of a recently synthesized extracellular matrix, called osteoid. Osteoid, composed primarily of type 1 collagen, proteoglycans, and water, matures over time, deposits hydroxyapatite crystals, and then achieves the stability needed for loading a potential implant [18]. This process is largely dependent on macrophages, which secrete TNFα, one of the most potent inhibitors of OBs differentiation from SSCs [42]. Bone remodeling is regulated by signaling pathways involving mechanical stimulation, parathyroid hormone (PTH), osteocalcin, and Wnt signaling [43]. The Wnt pathway is the most important process in SSCs differentiation into OB. The Wnt pathway encompasses a large group of signaling molecules that promote osteoblastogenesis and inhibit adipogenesis. Loss-of-function mutations in this pathway lead to an osteoporotic phenotype, whereas gain-of-function mutations support high bone mass, even in a nonphysiological state [18,43]. OBs are sensitive to PTH. This anabolic hormone secreted by the parathyroid gland promotes bone formation and induces the secretion of prohematopoietic factors such as IL-6, IL-6R, and MCP-1 by OBs [18]. In contrast, molecules such as sclerostin and Dickkopf proteins contribute to the inhibition of bone formation and the promotion of bone resorption under pathological conditions or without mechanical loading [18,43]. Osteocytes, functioning as mechanosensitive cells, are not an independent cell lineage type but rather the final stage of OBs development [35,43]. Transformation begins passively by locking OBs in a synthetic matrix [35]. This prevents mitotic divisions and significantly slows down their metabolism. However, when considering their function in the context of the entire bone tissue, they play a significant role by tightly adhering to each other [27]. They recognize stresses generated by fluid movement in the bone canal system. Through mechanotransduction, these stimuli are interpreted, and various factors are secreted in response [44]. The absence of mechanical loading leads to the secretion of FGF-23, RANKL, and paracrine sclerostin, which inhibit bone growth. FGF-23 additionally inhibits bone mineralization [18]. OCs mediate bone resorption [18,43]. In addition to bone destruction, this process mediates anabolic processes, as the newly degraded matrix can be rebuilt by OBs at the site of need. Therefore, ensuring bone strength depends on OBs and OCs [18]. Bone cell activity is tightly regulated by cytokines, growth factors, and direct intercellular communication [26,43]. Among the key signaling pathways involved in bone remodeling, the RANK and RANKL play a key role in OCs differentiation and activation [26]. Importantly, bone tissue is in constant interaction with the immune system through complex cellular and molecular mechanisms [18,26,45]. This relationship is also reflected in their developmental origin, as OCs are derived from hematopoietic stem cells of the monocyte–macrophage lineage [26,43], while OBs, as previously mentioned, are derived from MSCs [19,26,43]. Among the immune cells involved in peri-implant healing, macrophages are considered key regulators coordinating the transition between inflammation, tissue repair, and bone regeneration [18].

### 3.2. Cytokines and Signaling Pathways

**Figure 1:**
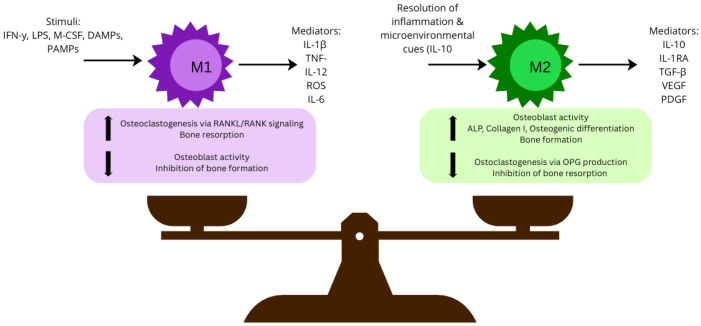
Osteoimmunology at the implant surface.

Macrophages play a central role in osteoimmunological processes occurring at the implant interface by regulating the balance between inflammation and tissue regeneration [25,46]. Immediately after implantation, the biomaterial surface interacts with blood components and immune cells, triggering an acute inflammatory response necessary for pathogen elimination, removal of damaged tissue, and initiation of wound healing [16]. During this early phase, macrophages predominantly acquire the pro-inflammatory M1 phenotype under the influence of stimuli such as interferon gamma (IFN-γ), lipopolysaccharide (LPS), and macrophage colony-stimulating factor (M-CSF) [47]. M1-polarized macrophages release pro-inflammatory cytokines including IL-1β, TNF-α, and IL-12, as well as reactive oxygen species, thereby promoting pathogen clearance and activation of Th1-mediated immune responses [47]. This transient inflammatory environment is essential for initiating tissue repair and recruitment of regenerative cells. However, excessive or persistent M1 activation may result in prolonged inflammation, foreign body reactions (FBR), delayed healing, and impaired osseointegration [25,40]. Sustained production of pro-inflammatory mediators additionally enhances osteoclastogenesis and peri-implant bone resorption through activation of osteoimmune pathways such as the RANKL/RANK signaling axis [23,26]. Successful implant integration, therefore, requires a gradual transition toward the anti-inflammatory and pro-regenerative M2 macrophage phenotype [25,47]. M2 macrophages produce cytokines including IL-10, IL-1 receptor antagonist (IL-1RA), and transforming growth factor β (TGF-β), which contribute to inflammation resolution, extracellular matrix remodeling, angiogenesis, and tissue regeneration [47,48]. In particular, M2c macrophages stimulated by IL-10 promote tissue remodeling and suppress excessive inflammatory activity through secretion of IL-10 and TGF-β [47,48]. Macrophages also directly regulate bone metabolism through interactions with MSCs and OB precursors [25,46]. Co-culture studies involving bone marrow stromal cells and monocyte/macrophage lineages demonstrated enhanced alkaline phosphatase activity, increased collagen type I production, and stimulation of osteogenic differentiation [25]. Activated macrophages may additionally promote bone formation through the secretion of osteogenic mediators such as oncostatin M [46]. Furthermore, macrophage-derived environmental signals support vascularization of regenerative biomaterials, which is essential for stable osseointegration [25,46]. Therefore, successful osseointegration depends not on the absence of inflammation itself, but rather on its precise temporal regulation and resolution. A balanced transition between M1 and M2 phenotypes is essential for maintaining FBE and promoting long-term implant integration, whereas disruption of this balance may lead to chronic inflammation, peri-implant bone loss, and implant failure [16,25,40]. The mechanisms behind osteoimmunology at the implant surface are shown in Figure 1.

### 3.3. Cytokines and Osteoimmune Signalling Pathways

The RANKL/RANK/OPG axis represents one of the principal regulatory systems controlling osteoclast differentiation and activation, and therefore plays a fundamental role in maintaining the balance between bone formation and bone resorption [23,26]. RANK is a type I transmembrane receptor belonging to the TNF receptor superfamily and is highly expressed on osteoclast precursors and mature osteoclasts. Its ligand, RANKL, is primarily produced by OBs and activated T lymphocytes and occurs in both membrane-bound and soluble forms [26]. Binding of RANKL to RANK initiates intracellular signalling cascades involving TNF receptor-associated factor 6 (TRAF6), p38 mitogen-activated protein kinase (MAPK), and activation of nuclear factor κB (NF-κB), ultimately leading to transcription of genes associated with osteoclast differentiation and activation [26,49]. NF-κB additionally serves as a major regulator of inflammatory signalling and both innate and adaptive immune responses [49]. Activation of these pathways within cells of the monocyte–macrophage lineage contributes to osteoclastogenesis, peri-implant bone resorption, and progression of inflammatory peri-implant diseases [23,25,26]. Osteoprotegerin (OPG), produced mainly by OBs, functions as a soluble decoy receptor for RANKL. By competitively binding RANKL, OPG inhibits its interaction with RANK, thereby suppressing osteoclast formation and activity [26]. Consequently, the RANKL/OPG ratio is considered a critical determinant of bone remodelling dynamics, with elevated ratios favouring bone resorption and inflammatory bone destruction [26]. The biological effects of macrophage polarisation are closely linked to activation of these osteoimmune signalling pathways. Persistent pro-inflammatory conditions characterised by excessive M1 macrophage activity promote RANKL expression and osteoclast activation. In contrast, resolution of inflammation and predominance of anti-inflammatory mediators support bone regeneration and stable osseointegration [24,25,26,46].

## 4. Immune Response to Dental Implant Materials

### 4.1. Foreign Body Reaction and Healing Phases

**Figure 2:**
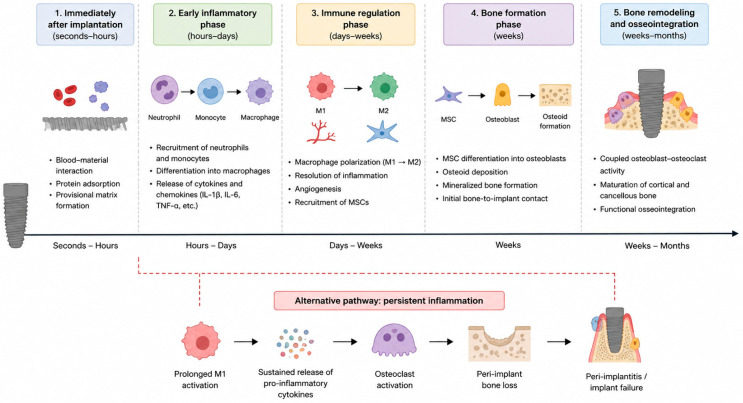
Immune reaction to foreign bodies.

After implantation of a dental implant, the biomaterial surface rapidly interacts with blood components and extracellular matrix distributions, which are used for adsorption and a functional response [16,50]. This early host response is a critical step that occurs when osseointegration or inflammation occurs. Neutrophils, monocytes, and macrophages are recruited to the implant interface, releasing cytokines and chemokines that later control bone repair and remodeling [8,16]. Bone healing around implants occurs through an overlap of bone formation and remodeling processes [8]. In these wireless modules, they interact with MSCs and osteogenic cells in the cancellous bone. MSCs differentiate into OBs, which are used to produce mineralized bone matrix, and are included in ten methods of new bone formation around the implant [16]. For the implant to connect to the bone and implant, new bone formation is necessary in the form of cortical and cancellous bone. Cancellous bone consists of bone barrels interspersed with bone marrow outlets, which serve as a reserve for MSCs. These cells are responsible for maintaining bone mass stability throughout life. They are a single, ready-to-differentiate osteoblastic generator, which is exclusively responsible for secreting bone matrix. Various factors, including mechanical stress, aging, and metabolic diseases, affect the fate of progenitors and stem cells. This process may be the solution or solution to osseointegration at the bone-implant interface [9]. Mechanical stress, aging, metabolic disturbance, and local inflammation can determine the regenerative capacity of these progenitor cells and, consequently, the effect of osseointegration [12,16]. Proper resolution of the early reaction is necessary for implant initiation. Once healing is initiated, it is a transient inflammatory state; unstable or recurrent inflammation can impair access and increase the risk of bone loss around the implant and its source [12,41]. As discussed in Section 3.2, macrophage polarization and signaling pathways, osteo, a key function in regulating the balance between inflammation and regeneration. Immune reactions have been summed up in Figure 2.

### 4.2. Immune Regulation of Osseointegration

Age is considered one of the major risk factors for implant failure. Advanced age is associated with compromised local bone conditions, prolonged healing capacity, and a greater likelihood of systemic health alterations, all of which may negatively affect implant outcomes. Consequently, the risk of implant failure tends to increase with age [12,51]. Sex-related differences in implant outcomes may also be associated with hormonal influences on bone metabolism. Estrogen deficiency, particularly in postmenopausal women, has been linked to increased bone resorption and impaired bone remodeling, potentially affecting osseointegration and long-term implant stability [43]. In addition to patient-related factors, the biological response to dental implants is strongly influenced by the physicochemical properties of the implant material itself. Surface roughness, hydrophilicity, chemical composition, and topography affect protein adsorption, immune cell adhesion, macrophage polarization, and ultimately the quality of osseointegration [16]. Contemporary evidence indicates that implant materials are not biologically inert but actively modulate the local immune microenvironment at the implant interface [16,41]. Titanium, currently the most widely used dental implant material, is generally considered highly biocompatible. However, titanium particles and ions released during implantation or long-term mechanical wear may contribute to peri-implant inflammation and disruption of mucosal homeostasis [22,24]. Furthermore, implant surface characteristics influence macrophage polarization and the balance between pro-inflammatory and anti-inflammatory responses. Surface modifications that promote M2 macrophage polarization have been associated with enhanced angiogenesis, bone regeneration, and improved osseointegration, whereas persistent M1 activation may favor chronic inflammation and peri-implant bone loss [25,46]. Consequently, modern implant design increasingly focuses on the development of immunomodulatory biomaterials capable of promoting favorable osteoimmune responses and long-term implant stability.

### 4.3. Link to Peri-Implant Diseases

Recent research has focused primarily on the molecular biology, genetics, and cell biology involved in periimplantitis. According to recent studies, the key proinflammatory cytokines affecting bone and responsible for periimplantitis include IL-6, IL-1β, IL-17, MMP, RANKL, and TNFα. In contrast to pro-inflammatory factors, cytokines such as IL-10, IL-1RA, OPG, TIMPs, and SERPINs inhibit osteoclast activation and the resulting bone destruction [21].

Among pro-inflammatory mediators, IL-6, IL-1β, IL-17, and TNF-α play central roles in the initiation and maintenance of peri-implant inflammation. Elevated levels of these cytokines have been consistently detected in peri-implant crevicular fluid (PICF) and are associated with disease severity, increased probing depth, bone resorption, and implant failure [21,52,53,54]. TNF-α and IL-1β are particularly important due to their ability to promote osteoclastogenesis and amplify the inflammatory cascade.

Matrix metalloproteinases, especially MMP-8 (aMMP-8), are key mediators of extracellular matrix degradation and connective tissue destruction. Increased levels of MMP-8 correlate with osteolysis and peri-implantitis severity, making it one of the most promising biomarkers for disease diagnosis and monitoring. MMP-9 has also been linked to progressive bone loss, although the exact interactions between different MMPs remain incompletely understood [21,55,56,57].

In contrast, anti-inflammatory mediators contribute to maintaining immune homeostasis at the peri-implant interface. IL-10 suppresses excessive inflammatory responses, while IL-1RA competitively inhibits IL-1 signaling, thereby limiting tissue damage and supporting osseointegration [21,53,54,58,59]. Additionally, SERPIN family proteins have emerged as potential biomarkers of peri-implantitis, as their expression is increased in diseased tissues and positively correlates with inflammatory cytokine levels. Together, these mediators reflect the dynamic balance between pro-inflammatory and protective mechanisms that determine the progression of peri-implant disease. Major inflammatory mediators associated with peri-implantitis are summed up in Table 1.

## 5. Material Properties Shaping Immune Modulation

### 5.1. Bulk Material Composition

The term successful osseointegration refers to the direct structural and functional connection between living bone tissue and the surface of an implant [9,60]. Over time, implantology has shifted away from materials that are merely bioinert, toward those that exhibit bioactivity and can modulate immune responses [7]. The emerging discipline of osteoimmunology emphasizes that bone formation and maintenance result from a continuous, two-way communication between bone cells and immune cells [7,9].

**Figure 3:**
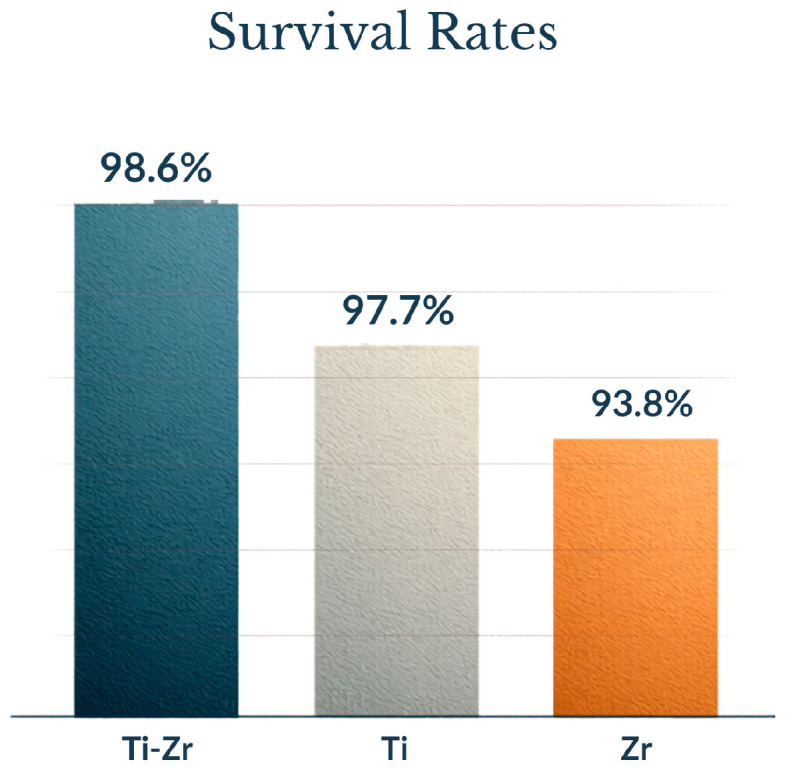
Comparative clinical performance and mechanical properties of dental implant materials.

Titanium (Ti) remains the standard choice in dental implants mainly because it combines high predictability, good biocompatibility, and favorable mechanical properties [3,7]. Still, its grayish appearance and possible metal ion release have prompted interest in zirconia (Zr)—a bioinert ceramic—as an alternative, especially for patients with thin gingival tissue. Studies generally show that Zr implants integrate with bone similarly to Ti, though meta-analyses indicate slightly lower survival rates for Zi implants (93.8%) as opposed to Ti’s 97.7% [3,5]. To merge titanium’s strong tensile properties with better biocompatibility, titanium–zirconium (Ti-Zr) alloys were developed. These alloys show the highest survival rates—about 98.6% [3,6]. Specifically, research clarifies these clinical outcomes by reporting survival rates of 98.6% for Ti-Zr, 97.7% for Ti, and 93.8% for Zr implants, highlighting the statistically lower performance of the Zi group compared to titanium-based materials [3]. Compared to commercially pure Ti, Ti-Zr alloys boast roughly 40% greater tensile strength, making them particularly suitable for narrow diameter implants (NDIs) used in regions subject to significant load, which improves cost-effectiveness by reducing the need for invasive bone augmentation [6]. Survival rate comparison in Figure 3 demonstrates that Ti-Zr alloys exhibit the highest clinical success (98.6%), followed by commercially pure Ti, 97.7%, while Zr implants show statistically lower survival (93.8%).

### 5.2. Surface Topography and Roughness

Surface topography plays a key role in shaping the immune microenvironment that surrounds an implant [7,28]. It has been demonstrated that microrough surfaces (Sa 1.0–2.0 μm) generally lead to better osseointegration than smooth surfaces (Sa 0.0–0.4 μm) because they promote the adhesion and differentiation of osteoprogenitor cells [7,9]. Conventionally, surface modifications aim to induce contact osteogenesis, which refers to the growth of bone directly on the implant surface. However, topography also affects distance osteogenesis, which initiates from the surrounding bone walls, partly through the activity of innate immune cells [9]. Contemporary biomimetic surfaces often present hierarchical micro- and nanoscale features that resemble the natural resorption pits created by OCs [7]. These surface details seem to influence macrophage behavior, shifting them from a pro-inflammatory M1 state toward a pro-healing M2 phenotype, which in turn supports both bone formation and new blood vessel growth [7,9,28]. Quantitatively, conditioned media from these M2 macrophages have been shown to boost the migration rate of bone marrow mesenchymal stem cells (BMSCs) by nearly fourfold [28]. On the molecular level, these micro–nano hybrid structures activate signaling pathways, including YAP/Piezo1/AKT/ERK, which coordinate the secretion of osteogenic factors like BMP-2 and Oncostatin M [6]. However, surface roughness may also impact bone resorption indirectly; surfaces with moderate roughness (Sa range of 1.0–2.0 μm) can elevate the OPG/RANKL ratio in OBs, which tends to suppress excessive OC activity [7]. However, surface roughness can sometimes upregulate pro-inflammatory cytokines (IL-1β, IL-6) under LPS conditions [14]. Figure 3 illustrates the signaling pathways activated by implant surface characteristics. Smooth surfaces (Sa 0.0–0.4 μm) typically promote a pro-inflammatory M1 macrophage phenotype, characterized by secretion of cytokines such as TNF-α and IL-6. Hierarchical micro–nano topography (Sa 1.0–2.0 μm) activates the Piezo1 channel as an upstream mechanosensor. This triggers calcium influx (Ca^2+^) and downstream activation of the YAP/AKT/ERK signaling axis, promoting an elongated M2 pro-healing phenotype. M2 macrophages subsequently release osteogenic and angiogenic factors, including BMP-2 and Oncostatin M.

### 5.3. Surface Chemistry and Hydrophilicity

The chemistry of an implant’s surface largely dictates its initial interactions with host proteins [7]. A key factor here is hydrophilicity, a specific state of surface wettability that significantly influences the early immune response. Surfaces with higher hydrophilicity, characterized by a water contact angle of 0°, often show reduced carbon content (~20%) in their oxide layers compared to hydrophobic ones (>25%) [7,29]. Hydrophilic microrough surfaces can dampen inflammatory responses of neutrophils and dramatically lessen the formation of NETs—those web-like DNA structures linked to fibrotic complications and poor wound healing [7,9]. Additionally, increased wettability seems to steer the adaptive immune response toward a Th2/Treg phenotype, associated with healing and faster inflammation resolution, as well as greater recruitment of stem cells [7]. To achieve such effects, biomimetic coatings like nanohydroxyapatite (nHA) are applied to create nanostructured, hydrophilic surfaces that imitate the bone extracellular matrix, offering ample anchoring sites for bone cells [61].

### 5.4. Mechanical Properties and Mechanotransduction

The mechanical environment at the implant-bone interface dictates cellular fate through mechanosensing [5,7]. Although Ti is a stiff substrate, the topography influences how cells perceive the surface mechanical cues [7]. The Piezo1 channel acts as an upstream mechanosensor that mediates calcium influx and downstream activation of YAP signaling in response to surface topography [28]. Initial mechanical engagement, or primary stability, is a prerequisite for osseointegration [61]. Despite these advances, negative outcomes must be noted, such as the risk of mechanical delamination of plasma-sprayed coatings during insertion, which triggers unintended inflammatory reactions [61]. Furthermore, the presence of bacterial biofilms can disrupt intended immunomodulation by causing persistent neutrophil activation, overriding pro-healing cues [7]. Surgical techniques like osseodensification can improve this stability in low-density bone by compacting and autografting bone particles at the interface, thereby enhancing early bone-implant contact [61]. The synergy between bulk material chemistry, surface topography, and mechanical properties determines the success of an implant [7,9,29]. By engineering surfaces that favor M2 macrophage polarization and reduce pro-inflammatory neutrophil activity, clinicians can better control the “Ally or Enemy” status of the immune system to ensure long-term osseointegration [7,28].

The topography of the implant surface is a key factor in the long-term osseointegration success of the dental implant [28]. The topography of the implant surface affects the behavior of progenitor and immune cells, their interactions, and the bone formation that follows. Modulating immune responses by engineering Ti surfaces may lead to improved osseointegration in healthy and compromised environments. Evidence from multiple experimental scales, including in vitro and in vivo studies, indicates that implant surface topography directly regulates osseointegration via modulation of the osteogenic activity of adherent MSCs and osteoprogenitor cells [7]. In recent years, Ti with meso-, micro-, and nano-scale roughness has been shown to significantly enhance osteoconductive and osseointegrative ability, while also modulating the phenotypic response of adherent macrophages. Micro–nano surface topography of Ti disks results in triggering M2-type polarization of macrophages. Macrophages activated by micro–nano surface topography Ti disks enhance osteogenic differentiation of BMSCs and angiogenesis of human umbilical vein endothelial cells. Micro–nano surface topography of Ti implant reduces inflammatory response in the alveolar bone defect model [28].

## 6. Immunomodulatory Surface Engineering Strategies

Various modifications of the bioactive surface of titanium dental implants are planned to improve the osseointegration process and prolong the implant’s resistance in the bone. According to new research, bioactivity modification has the ability to induce an appropriate biological response, which leads to increased bone-implant contact (BIC). Hydroxyapatite (HA) and its combinations with type I collagen or BMP-2 indicated a positive effect, as well as bioactive glass, growth factors, and nanostructured coatings containing calcium or calcium phosphates. Those nanostructures promote tissue mineralization, bone healing, and improve proper load distribution during implant use [25,62].

Research has shown that topographical modification of the HA surface via nanostructuring significantly increases new bone formation around the implant and the BIC value when compared to unmodified titanium surfaces used as a control group. The additional use of type I collagen in the coatings accelerates the healing process and reduces the activity of macrophages and OCs. However, the collagen coating alone is not sufficient [9,16,63].

Extended research has shown that BMP-2 and its potential to increase osteogenic responses on the implant surface, particularly in combination with nanostructured carriers. Research demonstrated that implants modified with BMP-2 and nHAp exhibited greater bone-to-implant contact (BIC) and a stronger osteogenic response in vivo when compared to titanium implants treated with resorbable blasted media, which served as the negative control group. Better bone formation occurs when BMP-2 is delivered by way of a nano-hydroxyapatite (nHAp) compared to unmodified control surfaces without BMP-2 enrichment. Additionally, BMP-2 incorporation was associated with improved recruitment and specialization of MSCs for osteoblastic lineages, contributing to the early stages of osseointegration. Despite these positive findings, it was also observed that the overall benefits of BMP-2 depend on medium properties and delivery kinetics, highlighting the need for further research on this method [14,46,63].

For effective osseointegration and to maintain a balance between bone formation and resorption, the use of implant systems with sustained-release ions is also essential [37]. Bioorganic metal ions such as magnesium (Mg), strontium (Sr), silicon (Si), zinc (Zn), and copper (Cu) play a key role in bone metabolism and remodeling [2,31].

Mg supports the bioactive properties of biomaterials by stimulating cell adhesion, proliferation, and differentiation, which promotes bone regeneration [31]. In addition, nanostructured Sr coatings can increase the expression of genes associated with osteogenesis and reduce markers associated with OCs activity, leading to enhanced OBs differentiation and reduced bone resorption [13,32]. Coatings based on materials such as SiC and SiCN have good biocompatibility with OBs. The strongest are observed on surfaces coated with QSiCN. Titanium surfaces containing copper (TiCu) help stabilize the balance between aerobic and anaerobic bacteria in the implant environment and saliva, which is essential for the health of peri-implant tissues [33].

Dental implants can also be coated with various medications. It is maintained that substances such as tetracycline, vancomycin, bisphosphonates, and simvastatin effectively eliminate bacteria, promote cell proliferation, and support bone repair. In addition, coatings containing antimicrobial peptides (AMPs) exhibit a wide spectrum of activity against various pathogens. These include, for example, very helpful bactericidal peptides, including GL13K and human beta-defensins (HBDs) [10,26,47].

In the context of historical development and foundational concepts, the concept of OIM was defined as a key parameter for evaluating bone biomaterials, indicating that surface design must actively modulate the immune environment rather than merely remaining biologically inert [15]. Additionally, research highlights the fundamental role of angiogenesis in implant dentistry, pointing to the necessity of combining surface topographical features with the promotion of vascularity to achieve clinically friendly osseointegration [64]. Furthermore, it was demonstrated that the FBR is an inherent part of the osseointegration process, and its proper control through surface engineering determines the permanence or failure of the implantation [17]. Studies also emphasized that traditional biofabrication methods often fail to account for the complexity of material-host interactions, requiring new approaches in bone tissue engineering for successful translation.

Regarding the clinical application of these various materials, safety remains a primary concern. The use of bioactive elements like Zn, Mg, or Si represents an attractive alternative to biomolecules due to their higher stability and reduced biotic risk or immunogenicity. While traditional designs sought inert materials, clinical evidence now forces a shift toward materials that “reprogram” the immune system. However, in the case of synthetic polymers, prophylactic drug treatments may be necessary to avoid complement-mediated reactions. Survival rates of implants modified with these strategies are highly encouraging; meta-analyses of clinical data indicate a high 10-year survival rate for dental implants at 96.4% [4]. Specific systems, such as Dentium, have achieved a cumulative survival rate of 97.37% after 5 years [65]. In terms of material performance, titanium remains the clinical standard due to its excellent mechanical properties and biocompatibility. While Sr-doped materials show significant improvements in bone density—even in osteoporotic conditions—care must be taken with bioactive glasses, as their flexural strength can be lower than that of cortical bone, potentially limiting their use in high-load areas. From a cost-effectiveness perspective, utilizing trace elements (Ca, Cu, Zn, Mg) is far more economical than expensive and unstable growth factors or unique extracellular matrix (ECM) proteins.

Current challenges in the field must also be addressed, particularly long-term material stability. Delicate nanocoatings may be damaged during surgical insertion, leading to the release of wear particles that can induce chronic inflammation and aseptic loosening via the NLRP3 inflammasome. Biofilm formation also remains a major cause of failure; the “race for the surface” means microbial biofilms often form faster than host tissues can integrate. Surfaces with increased roughness may inadvertently increase bacterial adhesion, necessitating strategies that prevent pathogen colonization while promoting integration. Finally, the development of three-dimensional tissue models is essential to overcome the limitations of traditional 2D in vitro models. Future research should prioritize advanced platforms such as bone organoids and organ-on-a-chip systems that closely mimic the structural and functional microenvironment of native bone, enabling more predictive testing before clinical implementation.

## 7. Nanotechnology and Smart Implant Surfaces

The development of antibacterial–osteogenic dual-functional surfaces is a critical advancement in dental implantology, aimed at overcoming the causes of treatment failure, such as the negative capability of surface-forming osseointegration and post-operative infection [34]. Titanium implants possess excellent biocompatibility and mechanical strength; however, they lack inherent antibacterial properties, making them susceptible to biomedical device-associated infections (BAIs) [37]. Emerging multifunctional surfaces are designed to inhibit bacterial colonization and promote stable bone-to-implant contact. These functions can be integrated into dental implant surfaces by several innovative approaches [34]. Modification of dental implant surfaces is emerging to achieve long-term success in therapy by stimulating cell bioactivity and enabling bactericidal functions [1].

Modifications of dental implant surfaces are connected to their nanostructure. The most widely researched nanostructures for dental implants include nanotubes and nanopores. Nanotubes are hollow structures that are open at the top and closed at the bottom. Nanopores differentiate from nanotubes by having a fused top with a porous architecture [1]. Alternative morphologies of nanostructure, such as nanopillars, nanograss, or nanotemplates, have yet to be adequately studied for clinical use in dental implants. However, the bionanostructures are becoming more popular among researchers due to their excellent properties, such as superhydrophobicity, self-cleaning, and antibacterial osteogenic dual-efficacy. They enable osseo and soft-tissue integration and can contain local-releasing therapeutics [1]. Fabricated bionanostructures of cicadas using titanium nanopillars were found to disrupt bacterial morphology, reduce their adhesion, and promote adhesion of OBs. Surface nanostructures of other animals, such as dragonfly and butterfly wings, shark skin, and gecko feet, seem to have similar self-cleaning, bactericidal, and biocompatibility properties, which can be useful in implant surface modification [14].

**Figure 4:**
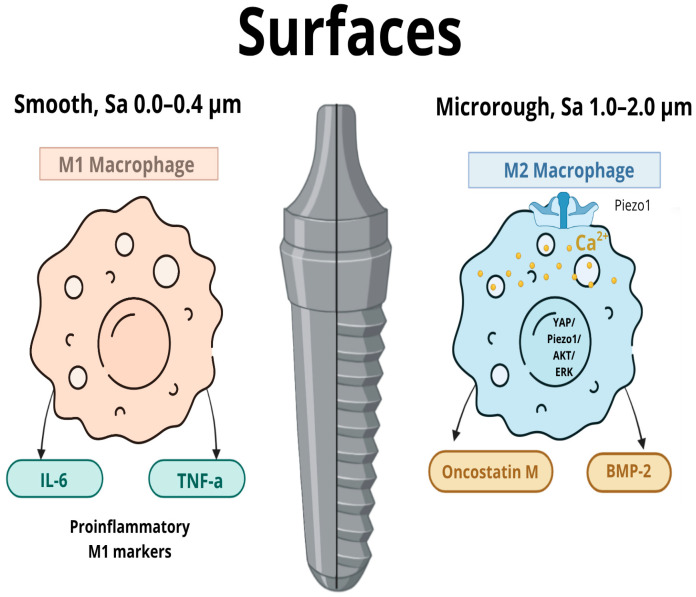
Illustration of surface-mediated immunomodulation and mechanotransduction.

**Figure 5:**
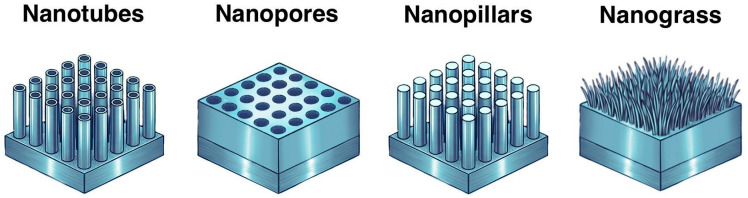
Types of dental implants: nanostructure.

Nanostructures directly influence the biological cascade required for successful osseointegration. Providing a high surface area alters the biochemical response, improving protein adhesion and cell behavior, but also helps achieve a stronger and more durable bond between the bone and the implant surface. By providing nanoscale anchoring points stimulate the migration and osteogenic differentiation of MSCs. During the initial stages of augmentation, nanotopography supports the formation of a fibrin-rich blood clot, the subsequent migration of fibroblasts and inflammatory cells [1]. Studies show cell elongation and the formation of clusters, which are beneficial for stable implant integration [39]. Another process is mechanotransduction; the specific dimensions of these nanostructures influence mechano-signal transduction, which can guide cell spreading and attachment strength [1]. According to researchers, the sharp edges of the nanostructure can stretch and rupture the bacterial cell wall, causing bacteria to lyse [37]. This is especially effective against Gram-negative microbes, which are more sensitive to these nanostructures. Synergy of different surface types is noticeable with the microscale roughness of the titanium implant that enhances the contact-killing effect of the nanopatterns [34]. Surface-mediated immunomodulation and mechanotransduction are shown in Figure 4. Types of dental implants nanostructures are shown in Figure 5.

Implant surface treatments that are currently clinically utilized comprise machining, grid-blasting, acid-etching, electrochemical anodization (EA), plasma treatment, polymer coatings, TiO_2_ nanotube coatings (TNT), and UV photofunctionalization. Traditional grit-blasting employs the high-pressure bombardment of particles such as Al_2_O_3_, aluminum, or hydroxyapatite to impart micro- and nanoscale indentations. Acid-etching is often utilized to eliminate manufacturing residues and create roughened surfaces [1]. The combination of the mentioned methods (sand blasting and acid etching) is the Sandblasted Large-grit Acid-etched technique (SLA) [1]. SLA is clinically popular because studies have reported accelerating the orchestration of osseointegration within 1–2 months on the SLA implant surface [1]. However, Electrochemical Anodization (EA) is increasingly considered a superior strategy for fabricating precisely controlled nanostructures, such as TiO_2_ nanotubes and nanopores, on complex implant geometries [1]. Unlike other methods, EA is highly scalable and cost-effective, allowing for the fine-tuning of nanostructure dimensions to enhance both bone and soft-tissue integration [1]. Furthermore, EA can preserve the existing micro-roughness of an implant to create dual micro–nano structures, which provides a distinct clinical advantage over techniques like plasma treatment that carry a risk of coating delamination during surgical insertion [1]. Methods requiring additional coating of dental implant surfaces are plasma treatment, block copolymer coating, and TNT. A vacuum or low-pressure environment is used to coat implants with micro/nanoscale layers in plasma treatment. The result is an adherent layer on the dental implant surface, which, however, may break or delaminate during implant insertion. The use of this technique requires extreme surgical care. The nanostructured coatings made of poly(styrene-block-2-vinylpyridine) (PS-b-P2VP) act through a contact-killing mechanism. The polymer is dissolved in a solvent to form micelles, cast onto titanium substrates via spin coating, and then subjected to Solvent Vapor Annealing (SVA) to reorganize the molecules into specific patterns. The nanostructure depends on the type of solvent used during SVA. Usage of toluene results in the creation of micellar structures, while cylindrical features are generated in chloroform. Block polymer coating is highlighted as simple, cost-effective, and capable of coating large areas with precise control over nanotopography without requiring specialized or dedicated equipment [66]. TNT produced by oxidation increases the specific surface area of the implants and also improves their photoelectrochemical characteristics. This method promotes adhesion and proliferation of OBs through signaling pathways [14]. Beyond these mechanical and electrochemical methods, new approaches such as UV photofunctionalization emerge. Use of UV light in dental implant preparations transforms titanium surfaces from hydrophobic to super-hydrophilic, which increases OBs attachment and proliferation while simultaneously reducing the formation of bacterial biofilm [14]. Ultimately, the goal of these diverse modifications is to balance bioactivity enhancement with therapy such as bactericidal efficacy to ensure implant stability even in compromised patient conditions [34].

## 8. Advanced Manufacturing Approaches

The implant osseointegration can be improved by changing surface roughness, usually through machining, grid-blasting, acid-etching, EA, etc. However, the conventional methods used to modify the roughness of implant surfaces do not allow the fabrication of a structure with a completely controlled external shape design [38]. An alternative approach that can provide patient-specific shapes is AM, commonly known as 3D-printing. AM uses computer-aided design data to directly create a physical model. As opposed to traditional methods, 3D-printed implants can be made forthwith with a rough external structure. Consequently, they may not require post-processing treatment to modify the nanostructure of their surfaces. 3D-printed dental implants also perfectly fit a patient’s anatomy because the project file is created based on the patient’s radiographs and digital impressions [38]. AM processing allows the production of porous ceramic and metal structures. However, creating advanced high-strength dense ceramics is challenging with AM; the research on perfecting this process is growing rapidly. Techniques that use commercially available equipment are vat polymerization, direct ink writing, material jetting, and fused deposition of ceramics. All are capable of making ceramics with satisfactory strength parameters and desired surface structure [39]. To avoid defects of post-processed implant surfaces, such as delamination and microstructure variations, researchers created hybrid implants. Those structures are manufactured in a way that seamlessly combines a dense core with a porous surface layer. This AM implant has a unique surface with a directional lamellar pore morphology and also provides strength comparable to conventional Zr implants [39].

A promising method for manufacturing more efficient titanium implants is SLM. SLM is an AM technology that allows for precise control over pore size, shape, and interconnectivity. Structural characteristics are analogous to bone structure with similar elastic modulus and have osteogenic, angiogenic, and immunogenic properties [28]. 3D printed Ti implants are noted for their low stiffness, which has been found to enhance angiogenesis and osteogenesis [28]. This material helps regulate macrophage polarization through the Piezo1/YAP signaling axis, encouraging the immune system to support tissue repair [28]. Acid etching is used additionally to remove excess metal particles that are weakly adherent. By this process, the surface is more suitable for cell attachment and proliferation and has high anti-bacterial properties. Studies have reported that 3D-printed implants with porous structure improve implant osseointegration by promoting bone ingrowth [38]. Titanium surfaces manufactured through SLM can be modified to directly inhibit the differentiation of OCs by regulating the MAPK signaling pathway, which reduces bone-resorbing activity at the interface [7].

The success of 3D-printed implants is based on their osseointegration and immunomodulation properties. Research shows that porous surfaces manufactured through AM processing significantly enhance the proliferation of human oral OBs (hOBs) compared to conventional surface treatment; the growth rate was +38.76% ± 8.14% higher in comparison [38]. Topography of 3D-printed dental implants promotes the expression of critical osseointegration biomarkers, including osteocalcin (OCN), alkaline phosphatase (ALP), and BMP2. Significantly higher calcium deposition (+42.75% ± 8.91%) was observed on 3D-printed porous surfaces compared to machined surfaces [38]. 3D printing allows for the creation of hierarchical architectures that better mimic the native bone extracellular matrix [28]. This provides a potent mechanical cue to cells, which is critical for promoting the M2 polarization of macrophages and ensuring long-term functional outcomes [28]. In ceramic implants, the unique lamellar structure directs OBs to adopt an elongated morphology and uniform orientation. This alignment is associated with increased metabolic activity, proliferation, and enhanced long-term matrix mineralization [39]. Stable osseointegration is supported by angiogenesis. 3D-printed surfaces promote markers such as vascular endothelial growth factor and CD31 in MSCs. Furthermore, these surfaces favor the attachment of gingival fibroblasts (hGFs), which are essential for establishing a soft-tissue barrier and protecting the periodontium [38].

Immunomodulative components of AM-manufactured implants include macrophage polarization and genetic signaling. Studies on 3D-printed Ti surfaces indicate they can actively modulate the immune response by influencing monocytes and directing them toward the M2 (anti-inflammatory) phenotype, which is preparatory for tissue regeneration and angiogenesis [38]. Genetic Signaling is mediated by the increased expression of specific microRNAs (miRNAs) related to M2 macrophages, such as *miR-124*, *miR-130*, and *miR-483*, helping to create a pro-regenerative environment at the implant site [38].

Although porous structure tends to reduce the implant’s mechanical strength, hybrid dense-core designs help overcome that challenge. The effect of fatigue, together with the hydrothermal aging of ceramic implants, results in compression at the level just under the porous surface. Such pressure protects against implant fractures and prevents delamination of the porous zone [45]. Survival rate statistics show 94.3% to 97.8% success rate in different trials, which makes such implants extremely promising for dentistry rehabilitation [38].

## 9. Limitations and Future Perspectives

Nowadays, it can be observed that modern dental implantology is evolving toward an approach known as OII. In this model, treatment success no longer depends solely on the passive biocompatibility of the material, but rather on the implant’s ability to actively modulate the patient’s immune response. Clinical meta-analyses support this paradigm shift, reporting a robust 10-year survival rate for dental implants at 96.4%, with certain immunomodulatory systems like with certain immunomodulatory systems like Dentium (Dentium Co., Ltd., Suwon, South Korea) achieving 97.37% survival after 5 years [64].

In the field of immunomodulatory biomaterials, crucial aspects are modifications to the structure of dental implants, especially at the nanoscale. TiO_2_ nanotubes or nanospikes can influence the behavior of immune system cells, promoting regenerative processes and reducing inflammation [1,2,28]. At the same time, there is multiple research on the more frequent inclusion of multifunctional coatings containing metal ions such as Sr, Mg, or Cu. These modifications support bone formation, improve blood supply, and help maintain microbial balance in the oral cavity [6,13,31]. Innovative materials, such as QSiCN coatings, further enhance the adhesion of bone cells to the implant surface [36].

Concomitantly, the concept of personalized implantology appears as a vital futuristic approach. Thanks to technologies such as 3D printing and selective laser melting, it is possible to design implants tailored to the patient’s individual anatomy, with controlled porosity and surface structure [30,38]. In clinical practice, this translates to the possibility of using less invasive procedures, such as NDIs made of Ti-Zr alloys, which in many cases allow bone augmentation to be avoided [3,28].

From the dentist’s perspective, the development of these technologies primarily means greater treatment predictability, shorter procedure times, and reduced reliance on highly advanced surgical procedures. For the patient, this translates to faster healing, a lower risk of complications, and a shorter, less oppressive treatment process.

Moreover, the use of implants, capable of releasing active substances immediately after implantation, is being considered more and more frequently. Such solutions can support the healing process from the very first moments after surgery and reduce the need for additional interventions [1].

This part of dentistry is dynamically evolving, but analysis of the available literature reveals significant limitations and gaps in research. One of the main issues is that many studies are conducted using simplified models, such as flat Ti discs, which do not reflect the actual geometry of clinically used implants [1]. This means that the effectiveness of these solutions under real-world conditions may differ from what laboratory results suggest.

Another significant challenge is the durability and stability of surface modifications. Delicate nanocoatings may be damaged during implant insertion into bone, leading to the release of material particles and triggering unwanted inflammatory reactions [1,39]. This highlights the need to strike a balance between high bioactivity and the long-term mechanical stability of implants.

Another limitation is the insufficient representation of biological conditions in the research models used. Traditional 2D models do not account for the complex oral environment, including bacterial biofilms and the three-dimensional structure of tissues. Therefore, future research should focus on more advanced models, such as “organ-on-a-chip” systems or three-dimensional models [23,30], which recreate complex physiological microenvironments under controlled in vitro conditions. These biomimetic platforms facilitate the recapitulation of human organ physiology, narrowing the gap between in vitro assays and in vivo models by delivering the biochemical and biomechanical cues required to support authentic cell–matrix interactions. By providing a more faithful recreation of the native microenvironment through biological barrier interfaces, such as those based on collagen type I, these systems significantly enhance the predictive capacity of models used in biomedical research [67].

The clinical translation of advanced immunomodulatory nanocoatings is frequently challenged by the strict necessity of routine sterilization protocols, which can inadvertently compromise the engineered surface properties. It is well documented that various sterilization techniques can significantly alter the implant’s surface chemistry, hydrophilicity, nanotopography, and overall mechanical stability [1]. Specifically, high-temperature and high-pressure steam can degrade heat-sensitive biomacromolecular coatings and cause the structural collapse or delamination of delicate nanotopographies. Similarly, chemical sterilization using ethylene oxide (EtO) may alter the surface energy and wettability, while gamma irradiation—although highly effective for decontamination—can induce unintended chemical modifications, such as the crosslinking or oxidative degradation of collagen-based and polymeric layers [34]. Consequently, ensuring that the bioactivity and morphobiological integrity of these “smart” surfaces survive the sterilization and subsequent long-term storage processes remains a critical barrier that must be addressed before their widespread clinical implementation [1].

Despite promising research results, long-term clinical data confirming the durability and safety of many of these solutions for dental implants are still lacking. In particular, it is not fully clear how new materials and technologies affect the long-term risk of complications, such as peri-implantitis, so further research is necessary.

In conclusion, the design of dental implants has undergone a fundamental paradigm shift from achieving simple mechanical stability with bioinert materials to proactive OII [7,8,13,14]. This review demonstrates that the success of long-term osseointegration is dictated by the precise temporal regulation of the immune response, particularly the balanced transition of macrophages from a pro-inflammatory M1 to a pro-regenerative M2 phenotype [7,8,13,14,25,40]. By synergizing advanced surface engineering—such as hierarchical micro–nano topographies, bioactive ion doping (Sr, Mg, Cu), and antimicrobial coatings—with cutting-edge additive manufacturing, it is now possible to create “smart” implants tailored to the patient’s individual anatomy and physiological needs [1,2,7,10,13,14,26,28,31,32,33,36,38,39,47]. While significant challenges remain regarding the clinical stability of nanostructures and the need for more predictive 3D research models, the move toward immunomodulatory biomaterials offers a highly predictable pathway for enhancing implant survival, especially in patients with systemic risk factors [1,4,14,15,30,39].

Treatment strategies are summed up in Table 2.

## 10. Conclusions

The clinical success of dental implants no longer depends solely on achieving passive mechanical stability, but primarily on an early and precisely targeted response from the patient’s immune system. The transition from the traditional concept of the biological inertness of biomaterials to the paradigm of OII represents a fundamental step in the development of modern implantology. The accumulated evidence clearly indicates that the key factor determining the transition from the acute inflammatory phase to effective bone tissue regeneration is the early phenotypic switch of macrophages from the classically activated, pro-inflammatory M1 to the alternatively activated, pro-regenerative M2. A properly modulated immune microenvironment secretes a cascade of anti-inflammatory cytokines and growth factors that directly influence the activity of osteogenic cells by regulating signaling pathways, including the RANK/RANKL/OPG axis. This prevents the formation of fibrous tissue, limits excessive osteoclastogenesis, and protects against pathological bone resorption, which is the primary underlying cause of peri-implant diseases (peri-implantitis).

The design of modern implant surfaces has evolved toward proactive OIM. It has been demonstrated that advanced materials engineering strategies, such as precise modifications to micro- and nanotopography and increased surface hydrophilicity, can induce desired cellular behaviors at the level of mechanotransduction. Furthermore, innovative methods, including the doping of biomaterials with bioactive ions (such as strontium or magnesium) and the creation of smart coatings that release drugs and antimicrobial agents, exhibit synergistic, dual action—effectively inhibiting bacterial colonization while simultaneously stimulating OBs proliferation and angiogenesis. AM,3D printing, is also becoming a breakthrough in the field of bone reconstruction, enabling the creation of highly porous, biomimetic titanium and zirconium structures. These structures not only perfectly mimic the architecture and biomechanical parameters of natural bone but also promote an integrated response from circulating monocytes and MSCs.

Despite highly promising results from in vitro and in vivo studies, translating these technologies into everyday clinical practice remains hindered by significant research limitations. Much of the current knowledge is based on analyses conducted with simplified, two-dimensional models, such as flat titanium discs, which do not fully capture the complex, three-dimensional geometry of the threads on implants used in patients. Furthermore, the implementation of advanced nanostructured coatings requires further research into their mechanical stability and resistance to routine sterilization procedures, which may alter their physicochemical properties.

To fully realize the paradigm shift toward OII, future research must transition from evaluating static surfaces to developing dynamic, spatiotemporally controlled immunomodulators. A highly testable prediction is that next-generation “fit-and-forget” implants, engineered for the sequential release of therapeutic agents—such as an initial release of antimicrobial cues followed by a sustained release of M2-promoting factors like Polydeoxyribonucleotide (PDRN) or specific metallic ions (e.g., Sr^2+^, Mg^2+^)—will significantly outperform static titanium surfaces in compromised clinical scenarios.

Furthermore, we predict that specific 3D-printed hierarchical micro/nanotopographies can be tailored to actively target cell membrane mechanosensors, such as the Piezo1 channel, to predictably drive M2 macrophage polarization via the YAP/AKT/ERK signaling axis. Validating the efficacy of these advanced biomaterials will require abandoning simplified 2D cell cultures. Future studies must rigorously test these surfaces utilizing 3D bone organoids and microfluidic ‘bone-on-a-chip’ platforms that accurately replicate the dynamic biomechanical and immunological environment of the oral cavity. Ultimately, integrating data from these advanced biomimetic models with machine learning algorithms presents a testable framework to accurately predict patient-specific macrophage polarization, paving the way for truly personalized implant dentistry.

## Figures and Tables

**Table 1:** Major inflammatory mediators associated with peri-implantitis.

Cytokine/Marker	Function	Description	Ref.
IL-6	Pro-inflammatory and anti-inflammatory cytokines	Mediates inflammation, promotes osteoclastogenesis, is produced by osteocytes and macrophages; elevated in peri-implant crevicular fluid (PICF).	[21,52]
IL-1β	Pro-inflammatory cytokine	Central regulator of the inflammatory cascade; induces other inflammatory mediators; elevated in PICF and associated with implant failure.	[21,53]
IL-17	Pro-inflammatory cytokine	Produced mainly by Th17 cells; involved in immune signaling; levels correlate with probing depth in peri-implantitis.	[21,53]
MMP-8	Matrix-degrading enzyme	Degrades collagen types I and III; major mediator of tissue destruction; marker of peri-implantitis severity and osteolysis.	[21,57]
MMP-9	Matrix-degrading enzyme	Associated with bone resorption progression; may act synergistically with MMP-8 and MMP-13.	[21,57]
TNF-α	Pro-inflammatory cytokine	Associated with bone resorption progression; may act synergistically with MMP-8 and MMP-13.	[21,54]
RANKL	Osteoclastogenesis regulator	Promotes osteoclast differentiation and bone resorption; expression is enhanced by TNF-α.	[21,54]
IL-10	Anti-inflammatory cytokine	Produced mainly by monocytes; suppresses inflammation and contributes to immune balance around implants.	[21,53]
IL-1RA	Anti-inflammatory cytokine antagonist	Competitive inhibitor of IL-1α and IL-1β signaling; limits excessive inflammatory responses and supports osseointegration.	[21,53]
SERPINs (B1, B3, B4, B5)	Protease inhibitors	Inhibit protease activity; overexpressed in peri-implantitis; expression correlates positively with IL-6 and TNF-α levels.	[21]

**Table 2:** Summary of some representative treatment strategies with their mechanisms, effects, and limitations.

Treatment Strategy	Examples	Main Immune Mechanisms	Effect on Osteointegration	Limitations	Ref.
Surface Topography & Roughness	Microrough surfaces, hierarchical micro–nano structures	Triggers M2 macrophage polarization; activates YAP/Piezo1 signaling; elevates the OPG/RANKL ratio to suppress OCs	Encourages adhesion and differentiation of osteoprogenitor cells; enhances angiogenesis and bone formation	Laboratory models often use flat discs that do not reflect complex clinical geometries; nanostructures may be damaged during insertion	[6,7,9,28]
Surface Chemistry & Hydrophilicity	Super-hydrophilic surfaces, UV photofunctionalization, nHA coatings	Dampens neutrophil inflammatory response; reduces NETs formation; steers adaptive immunity toward a Th2/Treg phenotype	Faster resolution of inflammation; greater recruitment of stem cells; imitates bone ECM for better cell anchoring	Modern coatings may be sensitive to the high temperatures used during standard sterilization procedures	[7,9,29,61]
Bioactive Coatings	BMP-2, Type I Collagen, HA	Reduces macrophage and OC activity; improves recruitment and specialization of MSCs	Increases BIC; accelerates healing and tissue mineralization	BMP-2 success is highly dependent on medium properties and delivery kinetics; collagen alone is insufficient	[9,14,16,25,46,56,62,63]
Ion Doping	Sr, Mg, Cu, Zn	Sr stimulates the M2 phenotype while inhibiting OC activity; Mg supports bioactive properties and cell adhesion	Enhances OB differentiation; reduces bone resorption; Cu helps maintain microbial balance in the oral cavity	Possible release of material particles if coatings are damaged; potential concerns regarding metal ion release	[2,13,31,33,58]
Drug-Releasing Surfaces	Tetracycline, AMPs, bisphosphonates	Protects tissues from microbial invasion; minimizes inflammation by eliminating pathogens	Supports bone repair and cell proliferation; prevents BAIs	Requires precise control over mechanical stability and long-term release profiles	[10,26,47]
Nanotechnology	TNT, nanopores, nanopillars	Contact-killing mechanism: sharp edges stretch and rupture bacterial cell walls (lysis); provides nanoscale anchoring points	Improves protein adhesion and cell behavior; creates a stronger, more durable bond between bone and implant	Delicate nanostructures are susceptible to delamination or mechanical damage during surgical insertion	[1,14,34,37,39,66]
3D Printing	SLM, porous Ti/Zr structures	Regulates macrophage polarization via Piezo1/YAP signaling; increases expression of pro-regenerative miRNAs	Mimics native bone architecture; promotes bone ingrowth; enhances angiogenesis due to lower stiffness	Porous designs can reduce mechanical strength; high-strength dense ceramics are difficult to manufacture	[14,28,38,39]

## Data Availability

No new data were created or analyzed in this study. Data sharing is not applicable to this article.

## Abbreviations

The following abbreviations are used in this manuscript:

2D	Two-dimensional
3D	Three-dimensional
ALP	Alkaline phosphatase
AM	Additive manufacturing
AMPs	Antimicrobial peptides
BAIs	Biomedical device-associated infections
BIC	Bone-implant contact
BMP-2	Bone morphogenetic protein-2
Cu	Copper
EA	Electrochemical anodization
ECM	Extracellular matrix
EtO	Ethylene oxide
FBE	Foreign body equilibrium
FBRs	Foreign body responses
GCF	Gingival crevicular fluid
HA	Hydroxyapatite
HBDs	Human beta-defensins
hGFs	Gingival fibroblasts
hOBs	Human oral osteoblasts
IL-1RA	Interleukin-1 receptor antagonist
MAPK	Mitogen-activated protein kinase
Mg	Magnesium
miRNAs	MicroRNAs
MMPs	Matrix metalloproteinases
MSCs	Mesenchymal stem cells
NDIs	Narrow diameter implants
NETs	Neutrophil Extracellular Traps
NF-κB	Nuclear factor kappa B
nHA	Nanohydroxyapatite
nHAp	Nano-hydroxyapatite
OBs	Osteoblasts
OCN	Osteocalcin
OCs	Osteoclasts
OII	Osteoimmunological integration
OIM	Osteoimmunomodulation
OPG	Osteoprotegerin
PICF	Peri-implant cervical fluid
PDRN	Polydeoxyribonucleotide
PIM	Peri-implant mucosa
PS-b-P2VP	Poly(styrene-block-2-vinylpyridine)
PTH	Parathyroid hormone
RANK	Receptor Activator of Nuclear Factor κB
RANKL	Receptor Activator of Nuclear Factor κB Ligand
qPCR	Quantitative polymerase chain reaction
QSiCN	Quaternized silicon carbonitride
SERPINs	Serine Protease Inhibitors
Si	Silicon
SiC	Silicon carbide
SiCN	Silicon Carbonitride
SLA	Sand blasting and acid etching
SLM	Selective laser melting
Sr	Strontium
SSCs	Skeletal Stem Cells
SVA	Solvent Vapor Annealing
Ti	Titanium
Ti-Zr	Titanium–zirconium
TiCu	Titanium–copper
TiO_2_	Titanium dioxide
TNF	Tumor necrosis factor
TNT	TiO_2_ nanotube coating
TRAF6	TNF receptor-associated factor 6
Zn	Zinc
Zr	Zirconia
